# Needs assessment of people living with cognitive impairment/dementia, a requirement of comprehensive psychogeriatric assessment and person-centered care. Empirical validation of the model in a community study

**DOI:** 10.3389/fpsyt.2024.1481898

**Published:** 2024-12-17

**Authors:** Javier Vicente-Alba, Jesús Gutiérrez-Botella, Carmen García-Mahía, Raimundo Mateos

**Affiliations:** ^1^ Psychiatry Service, Vigo’s Health Area, Vigo, Pontevedra, Spain; ^2^ Psychiatry Department, University of Santiago de Compostela (USC), Santiago de Compostela, A Coruña, Spain; ^3^ Biostatech Advice Training and Innovation in Biostatistics Limited Society (SL) (USC), Santiago de Compostela, A Coruña, Spain; ^4^ Psychiatry Service, Coruña’s Health Area, A Coruña, Spain; ^5^ Psychogeriatric Unit, Psychiatry Service, University Complex Hospital of Santiago de Compostela (CHUS) University Hospital, Santiago de Compostela, Spain

**Keywords:** dementia, cognitive impairment, disability, dependency, needs assessment, comprehensive geriatric assessment, person-centered care

## Abstract

**Introduction:**

Cognitive impairment and dementia are part of a continuum that progressively leads to functional impairment and dependency. Dementia is a paradigmatic example of chronic and complex psychogeriatric diseases, requiring a comprehensive assessment. The authors underline the importance of implementing a formal assessment of needs (whether met or unmet) as an essential element of comprehensive assessment. The aim of this paper is to empirically validate this model of approach towards dementia, the needs assessment, demonstrating the relationship between needs and functionality/dependency in people with cognitive impairment/dementia in the community.

**Material and methods:**

Community-based, cross-sectional, descriptive epidemiological study based on the reanalysis of data from a two-phase community epidemiological study conducted in Santiago de Compostela, Spain, of 800 people over 65 years of age. The present study reanalyzes a subsample of 368 people, including those with dementia/cognitive impairment. The comprehensive assessment of the sample included sociodemographic variables, the presence of chronic diseases, health self-perception, assessment of affective, cognitive and functional state, as well as the needs assessment. The main instruments used were the MMSE, the Geriatric Depression Scale (GDS), the Barthel and Katz Indices, the Lawton Scale, and, for the needs assessment, the Camberwell Assessment of Need for the Elderly (CANE). For the clinical diagnosis of dementia, the ICD-10 criteria were followed. Statistical analysis: A study of the association between variables was carried out through hypothesis testing and a multivariate study was performed using regression models to analyze the relationship between the different variables defining disability/dependency, other health conditions and sociodemographic variables and the MMSE score as an expression of the cognitive impairment/dementia continuum.

**Results:**

People with cognitive impairment/dementia had a higher number of needs compared to the healthy population. The severity of cognitive impairment is a significant predictor of dependency in Basic Activities of Daily Living (BADL) and is also a predictor of a greater number of needs (both met and unmet).

**Discussion:**

The present study provides empirical evidence of the importance of implementing scales to assess the needs of people with cognitive impairment, as part of the process of comprehensive biopsychosocial assessment and person-centered care for dementia.

## 1 Introduction

The term cognitive impairment/dementia alludes to a continuum, a progressive and changing syndrome, which leads to successive disabilities and the loss of personal autonomy (i.e., to dependence on third parties) (1). Due to its enormous biological, psychological, and social complexity (both in terms of the patient and their caregiver), we consider dementia to be one of the best examples of complex chronic psychogeriatric diseases, and it will therefore always be at the center of reflection and psychogeriatric intervention. Geriatrics and Old Age Psychiatry both support a holistic approach as the essential tool to deal with this complexity (2, 3). In the health care field, the concept of need can be applied at both the collective and individual level (4). At population level, it serves as a tool to organize clinical management and design health policies. Likewise, the study of individual needs, once assessed and prioritized, allows for personalized interventions. The combination of decades of daily experience in the clinical approach to dementia and our research experience with CANE, an instrument used frequently to assess needs in psychogeriatrics, has led us to believe that needs assessment is an inextricable part of the comprehensive psychogeriatric assessment (5, 6). We are convinced that a care model based on a sufficiently thorough and operationalized study of the needs of the subject and their caregivers, while not essential, will greatly facilitate the comprehensive approach, the operationalization of the biopsychosocial model (more often advocated than actually put into practice), and, ultimately, person-centered care (7–10).

Models that assess the condition of older persons based on needs assessment take these aspects into account, identifying the need itself (understood as a deficit), the relevance or appropriateness of third-party assistance (whether from the family, the community or the health system) and how these needs vary over time. There are several theoretical models that integrate needs and their relationship with morbidity and disability, such as that of Miranda-Castillo et al (11), Shmidt et al (12) or the studies conducted by Kitwood (13). These models are not mutually exclusive; they share common points, while emphasizing different aspects. Thus, the Miranda-Castillo model relates the person’s needs to the clinical, social and caregiver spheres. Schmidt focuses on morbidity (in cognitive impairment) as the main element of loss of autonomy, adding the importance of knowing how the person copes with difficulties in maintaining their autonomy and what needs are most important to them. The latter idea is also emphasized in Kitwood’s publications, promoting person-centered interventions and, ultimately (along with previous models), highlighting the importance of meeting needs, relating them to autonomy, the state of well-being and quality of life.

With the aim of further examining and validating these theoretical models, a reanalysis of data from a community epidemiological study conducted two decades ago was carried out in order to empirically validate the needs assessment model, in this case, applied to people with cognitive impairment/dementia.

The aim of this study is to examine the relationship between needs and functional capacity/dependency in people with cognitive impairment/dementia, establishing the hypothesis that people with cognitive impairment will have a greater number of needs and a higher level of disability and dependency, and that the severity of cognitive impairment is in correlation with an increased number of needs (both met and unmet).

## 2 Materials and methods

This is a community-based, cross-sectional, descriptive epidemiological study of morbidity and other health-relevant conditions. It is based on a reanalysis of data from a community-based epidemiological study conducted in Santiago de Compostela, Spain, of people over 65 years of age (14, 15). It complies with the Strengthening the Reporting of Observational Studies in Epidemiology (STROBE) criteria (16).

The *sample* for the present study is composed of 368 subjects. The original study was a two-phase epidemiological survey. In the first phase, a community sample of 800 people over 65 years of age, representative of the Santiago-Barbanza Health Area, were interviewed in their homes and screened for cognitive impairment, depression and dependency.

The second phase included 368 older persons: a) those with suspected cognitive impairment and/or depression and/or physical problems leading to dependency (N=254), and b) a control subsample of people without cognitive impairment, depression or dependency (N=114). All the subjects in the second phase underwent a further interview to study their physical and mental health and needs, constituting the aforementioned subsample of 368 subjects included in the present paper.

The *sociodemographic variables* collected were: age, sex, level of education, marital status, cohabitation status (number of cohabitants, living alone or with a partner), profession, rural/urban environment.

The *clinical variables* collected in the research project (of which not all are presented in this paper) can be classified into: a) physical or mental morbidity variables; b) level of functionality/dependency.

The main *defining morbidity variables* in the project were: the Spanish 30-item version of the Minimental State Examination (MMSE) (17), the 30-item version of Yesavage’s Geriatric Depression Scale (GDS-30) (18), a self-referenced questionnaire of chronic diseases common in older persons (19) and a brief *ad hoc* questionnaire of Likert scale type questions on health self-perception. The clinical diagnosis of dementia was made according to the International Classification of Diseases (ICD-10) (20). The MMSE score was used to classify the level of cognitive impairment. Other clinical variables included the frequency of the subjects’ visits to their primary care physician/specialists and whether they had been hospitalized recently.

The Katz Index (21) was used to assess *functional level*, as well as the Barthel Index (22) (for the basic activities of daily living) and the Lawton & Brodie Scale (23) (for instrumental activities of daily living).

The *needs assessment* was carried out using the Camberwell Assessment of Need for the Elderly (CANE) (24, 25). This instrument analyzes 24 biopsychosocial needs of older persons and, if applicable, the caregiver’s overburden and need for information. This needs assessment is carried out from the triple perspective of the professional/researcher, of the caregiver (in the case of a dependent person) and of the subject themselves. For readers interested in gaining a deeper understanding of the instrument, we refer them to the two editions of the detailed manuals published on the subject (24, 25). It is worth noting that this is a semi-structured interview, in which “older people are fully involved in the needs assessment process and there is a special section noting their own views and their satisfaction with the amount of help received” (24). The professional (in this case, the researcher) bases their assessment of needs for each CANE item on the answers given by both the older person and their caregiver, also incorporating any contextual information from other research instruments. This approach allows each CANE item to be classified into one of three statuses: “no need”, “met need” or “unmet need”. The quantitative analysis of the CANE for a specific subject provides the total number of needs (differentiating between “met needs” and “unmet needs”). When group data are reported, the mean values of “met needs”, “unmet needs” and “total number of needs” are usually analyzed.

In the second phase of the study, 66 caregivers of dependent persons were also interviewed. They were assessed on: a) their level of social support; b) their level of stress; c) data on the cognitive impairment of the older person; d) perceived difficulties in their caregiving tasks and their coping strategies and, e) especially relevant for the present study, they filled out the CANE, providing their perspective of the needs of the older person in their care.

In the present paper, the quantitative data recorded in the professional/researcher column will be presented. At the *statistical* level, a study of the association between variables was carried out through hypothesis testing and a multivariate study was performed by means of regression models. Specifically:

1. For the association analyses between groups, Student’s t-test and χ^2^ tests were used to compare continuous and categorical variables, respectively.
2. For the multivariate analyses, since the data are count data with a large number of zeros, a generalized linear model with a negative binomial distribution was chosen. After fitting the complete model, a variable selection step was performed via the stepwise procedure, which consists in iteratively adding covariates to the model until the AIC (Akaike Information Criterion) metric is minimized. The best-fit model with the minimum number of covariates will have the lowest AIC (26).

In all cases, p<0.05 are considered statistically significant.

All the analyses included were performed in R version 4 (27).

At the *ethical and legal* level, the research data used were taken from the reanalysis of previous epidemiological studies, carried out between 1998 and 2000, accessed through anonymized databases. To carry out these studies, in accordance with the Spanish legislation in force at the time, verbal consent was requested from those interviewed and was approved by the corresponding Research Ethics Committee.

## 3 Results

The results of the study were organized under the following headings:

### 3.1 Description of the sample based on the presence or absence of cognitive impairment/dementia and sociodemographic data

**Table 1** shows the frequency distributions of the level of cognitive impairment and **Table 2** the sociodemographic variables (based on their sociological descriptive value, some variables that are not included in this publication have been presented).

**Table 1:** Sample subgroups according to cognitive impairment.

Variable	Values	Frequencies (% valid)
MMSE	No cognitive impairment (24≤MMSE ≤ 30)	109 (29.6%)
	Mild-moderate cognitive impairment (16≤MMSE ≤ 23)	203 (55.2%)
	Severe cognitive impairment (0≤MMSE ≤ 15)	47 (12.8%)
	Missing	9 (2.4%)
	Mean (SD): 20.6 (5.17) min < median < max: 1 < 21 < 30 IQR (CV): 7 (0.2)	

MMSE, Mini-Mental State Examination.

**Table 2:** Sociodemographic data of the sample.

Variable	Values	Frequencies (% valid)	Missing
Sex	1. Male	116 (31.5%)	0 (0%)
	2. Female	252 (68.5%)	
Age	Mean (D): 75.3 (6.8) min < median < max: 65 < 74 < 93 IQR (CV): 10 (0.1)		0 (0%)
Marital status	1. Single	50 (13.6%)	1 (0.12%)
	2. Married	158 (4.0%)	
	3. Widowed	155 (42.2%)	
	4. Separated	3 (0.8%)	
	5. DK/NA	1 (0.3%)	
Environment	1. Urban	59 (16.1%)	1 (0.27%)
	2. Rural	308 (83.9%)	
Cohabitation status	1. Alone	213 (57.9%)	0 (0%)
	2. With a partner	155 (42.1%)	
No. of cohabitants in the household	Mean (DT): 3.5 (2) min < median < max: 1 < 3 < 9 IQR (CV): 3 (0.6)	1: 60 (16.3%) 2: 91(24.7%) 3: 58 (15.8%) 4: 34 (9.2%) 5: 46 (12.5%) 6: 45 (12.2%) 7: 24 (6.5%) 8: 6 (1.6%) 9: 4 (1.1%)	0 (0%)
Level of education	1. Illiterate	41 (11.2%)	2 (0.54%)
	2. Reads/writes	258 (70.5%)	
	3. Primary education completed	58 (15.8%)	
	4. Secondary education/vocational training	3 (0.8%)	
	5. Higher vocational training	4 (1.1%)	
	6. University studies	2 (0.5%)	
Profession	1. Industrial/service entrepreneurs	0 (0.0%)	0 (0%)
	2. Merchants, self-employed owners	6 (1.6%)	
	3. Higher education, administration/business	1 (0.3%)	
	4. Secondary education, administration/business	6 (1.6%)	
	5. Clerical workers, sales representatives, auxiliary jobs	8 (2.2%)	
	6. Skilled workers, craftspeople	48 (13.0%)	
	7. Unskilled workers	45 (12.2%)	
	8. Farmers	217 (59.0%)	
	9. Sailors	6 (1.6%)	
	10. Not indicated	1 (0.3%)	
	11. Housewife	30 (8.2%)	
Type of pension received	1. Severe disability	2 (0.5%)	1 (0.27%)
	2. Full pension for their profession	2 (0.5%)	
	3. Non-contributory pension	16 (4.4%)	
	4. Social welfare fund	1 (0.3%)	
	5. Does not receive any benefits	13 (3.5%)	
	6. Retirement	231 (62.9%)	
	7. Total disability	10 (2.7%)	
	8. Widowhood	38 (10.4%)	
	9. Other	3 (0.8%)	
	10. NA	8 (2.2%)	
	11. Other types of benefits	43 (11.7%)	
No. of people dependent on the income	Mean (DT): 3.4 (2.8) min <median < max: -1 < 3 < 36 IQR (CV): 3 (0.8)		15 (4.08%)

### 3.2 Study of the relationship between functional capacity/dependency and the level of cognitive impairment/dementia

**Tables 3** and **4** show the contingency tables for the MMSE variable (cognitive impairment). The Barthel Index, the Lawton and Brodie scale and the Katz Index (**Table 3**), as well as the contrast of these variables, show a statistically significant relationship with these 3 scales (**Table 4**). People with cognitive impairment have a greater level of dependency, perceived as a worsening of the ability to perform basic and instrumental activities of daily living.

**Table 3:** Relationship between cognitive impairment/dementia and function/dependency (Barthel Index, Lawton and Brodie Scale and Katz Index).

Level of dependency/function according to the Barthel Index	Level of Cognitive Impairment (CI)		
	*No CI*	*Mild-moderate CI*	*Severe CI*
Independent	88	122	20
Mild dependency	20	76	14
Moderate-severe dependency	1	5	13
Level of dependency/function according to the Lawton and Brody Scale (L&B)	Cognitive Impairment (CI)		
	*No CI*	*Mild-moderate CI*	*Severe CI*
Independent	45	92	8
Mild dependency	16	36	7
Moderate-severe dependency	48	75	32
Level of dependency/function according to the Katz Index	Cognitive Impairment (CI)		
	*No CI*	*Mild-moderate CI*	*Severe CI*
A	91	137	21
B	14	45	11
C	2	10	1
D	0	6	1
E	1	2	2
F	1	2	6
G	0	0	5
H	0	1	0

**Table 4:** Results of the contrast of independence using the χ^2^ test for the dependency scales (Barthel Index, Lawton & Brody Scale, Katz Index) and the level of cognitive impairment/dementia.

Test	χ^2^ Statistic	Cramer’s V [95% CI]	P-value
Barthel (IB)	68.509	0.309 [0.228, 0.376]	< 0.001***
Lawton-Brody (ILB)	16.677	0.152 [0.057, 0.214]	< 0.01**
Katz (IK)	76.191	0.326 [0.219, 0.373]	< 0.001***

### 3.3 Study of the relationship between unmet needs and the level of cognitive impairment

With regard to the relationship between needs and cognitive impairment, three new variables were generated to assess needs: total number of unmet needs (those receiving the response “Severe Problem”), total number of met needs (those receiving the response “No problems due to the help given”) and the total number of needs (sum of met and unmet needs). **Table 5** presents a descriptive overview of these variables.

**Table 5:** Descriptive overview of the new variables to measure needs (CANE).

Variable	Values	Missing
Number of unmet needs	Mean (DT): 0.6 (1.4) min < median < max: 0 < 0 < 10 IQR (CV): 0 (2.4)	9 (2.45%)
Number of met needs	Mean (DT): 3.3 (2.4) min < median < max: 0 < 3 < 12 IQR (CV): 3 (0.7)	9 (2.45%)
Number of unmet + met needs	Mean (DT): 4 (3.1) min < median < max: 0 < 3 < 15 IQR (CV): 4 (0.8)	0 (0.0%)

**Figure 1:**
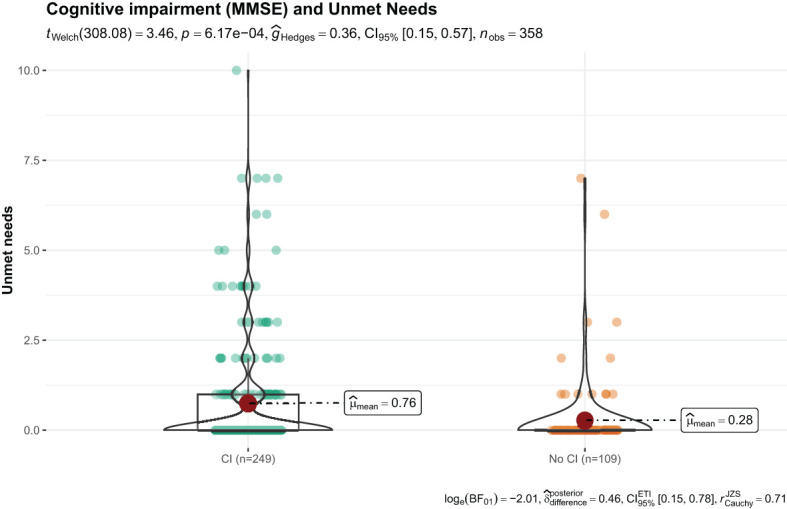
t-test results for unmet needs (based on CANE) and the measure of cognitive impairment (MMSE).

After analyzing the relationship between cognitive impairment/dementia and number of needs, a statistically significant relationship was found, with an increase in needs in individuals with cognitive impairment with respect to those without (**Table 6**, **Figure 1**).

**Table 6:** Results of the t-test comparing means of unmet needs based on CANE versus level of cognitive impairment/dementia.

Test	T-statistic	Mean differences (CI - no CI)	P-value
Cognitive Impairment (MMSE)	3.46	0.48	< 0.001***

#### 3.3.1 Multivariate analysis of statistically significant variables to search for predictors of unmet needs in relation to cognitive impairment/dementia

As indicated in the Material and Methods section, for the multivariate analysis a Generalized Linear Model of the negative binomial family with logarithm as the link function was performed to try to elucidate the important predictors in relation to the unmet needs response variable (count variable).

The following explanatory variables will be maintained:

• Depressive symptomatology: as a continuous variable, using the GDS scale.
• Cognitive Impairment: as a continuous variable, using the MMSE scale.
• Physical pathologies: as binary categorical variables for each disease.
• The only sociodemographic variable reported as significant: environment.

After verifying the presence of overdispersion in the data, mainly as a result of the excess of 0 values in the number of needs, this type of model was selected after comparing the fit offered compared to that of a Poisson-type model.

**Figure 2:**
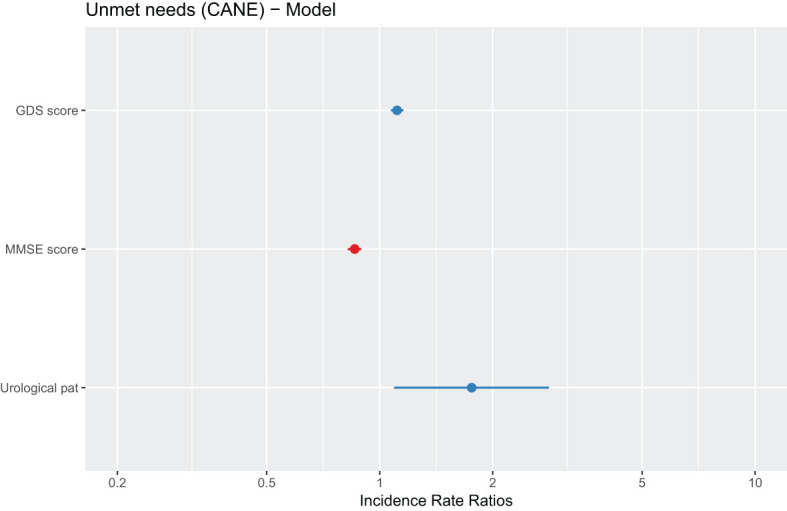
Graphical representation of the parameters estimated by the multivariate model. The dots represent the point estimate of the regression coefficient and the lines represent its standard error.

After making a selection of variables and eliminating those that are not significant, the final model includes the following predictors: GDS score, MMSE score and urological pathology (**Table 7**, **Figure 2**).

**Table 7:** Negative binomial regression model.

	Incidence Ratio	Standard Error	Z-statistic	P-value
(Intercept)	1.199	0.643	1.863	ns
GDS score	0.068	0.023	2.918	< 0.01**
MMSE score	-0.137	0.030	-4.551	< 0.001***
Urological Pat.	0.6213	0.295	2.102	< 0.05*

ns, not significant. Response variable: unmet needs based on CANE. Explanatory variables: pathologies and socioeconomic variables.

In this case, depression, dementia, and urological pathology are considered as significant predictors of the number of unmet needs.

• A higher value on the GDS depression scale is associated with a higher number of unmet needs.
• A lower value on the MMSE cognitive impairment scale (lower values indicate a greater severity of dementia) is also associated with a greater number of unmet needs.
• Having a urological pathology is associated with greater unmet needs.

### 3.4 Study of the existing relationship between met and unmet needs and the level of cognitive impairment

The relationship between pathologies and global number of needs (the sum of met and unmet needs) was also verified. The analysis followed the same procedure as that for unmet needs, now including met needs (total number of needs).

**Figure 3:**
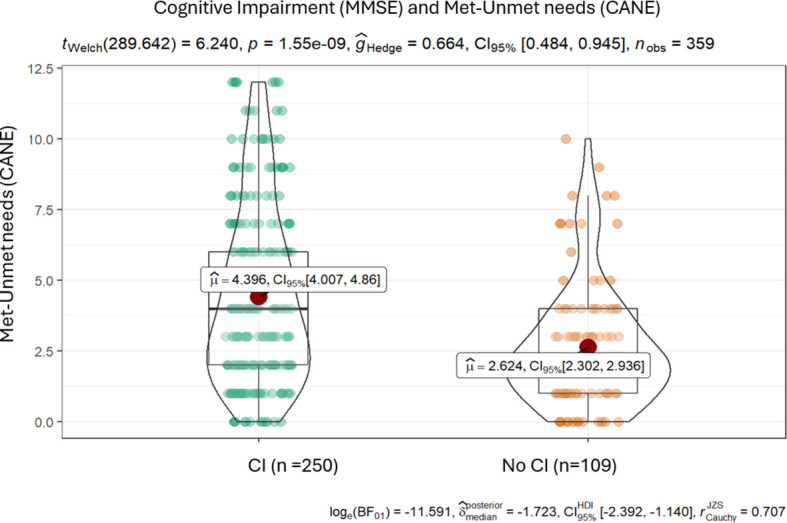
Results of the t-test for met and unmet needs (CANE) and the measure of cognitive impairment (MMSE).

The results are similar to previous ones; there are significant differences between healthy individuals and those affected by cognitive impairment/dementia or depression and, furthermore, these differences point to an increase in unmet needs in the latter (**Figure 3**).

The difference in means in the case of cognitive impairment is 1.8 (**Table 8**).

**Table 8:** Results of the t-test comparing means of met or unmet needs based on CANE versus the presence of cognitive impairment (MMSE).

Test	T-statistic	Mean difference (CI – no CI)	P-value
Cognitive Impairment (MMSE)	6.24	1.772	< 0.001***

#### 3.4.1 Multivariate analysis of statistically significant variables to search for met and unmet needs in relation to cognitive impairment/dementia

The multivariate model will be the same as that applied for unmet needs. The following explanatory variables will be maintained:

• Depressive symptoms: as a continuous variable, on the GDS scale.
• Cognitive impairment: as a continuous variable, on the MMSE scale.
• Physical pathologies: as binary categorical variables for each disease.
• Sociodemographic variables reported as significant: environment, sex, hospitalization, level of education and cohabitation status.

**Figure 4:**
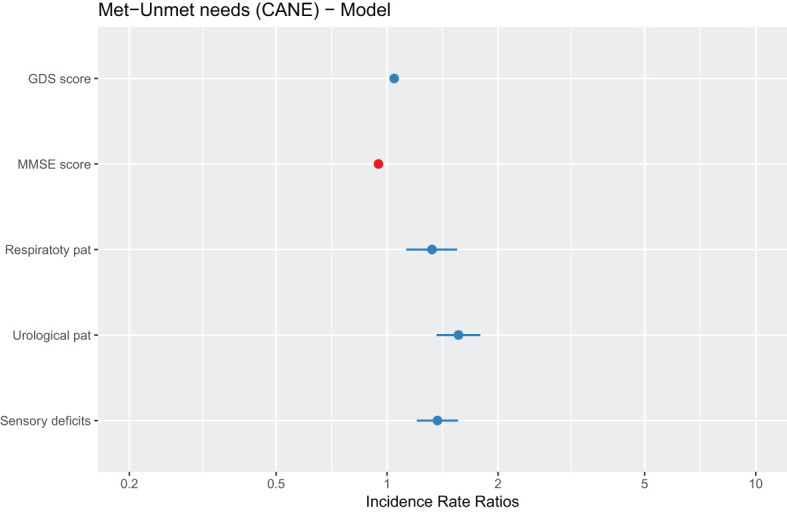
Graphical representation of the parameters estimated by the multivariate model. The dots represent the point estimate of the regression coefficient and the lines represent its standard error.

After making a selection of variables and eliminating those that are not significant, the final model includes the predictors shown in **Table 9** and **Figure 4**.

**Table 9:** Negative binomial regression model.

	Incidence Ratio	Standard Error	Z-statistic	P-value
(Intercept)	1.660	0.150	11.062	< 0.001***
GDS score	0.051	0.005	9.084	< 0.001***
MMSE score	-0.052	0.006	-8.069	< 0.001***
Respiratory pathology	0.222	0.082	2.696	< 0.01**
Urological pathology	0.428	0.077	5.513	< 0.001***
Sensory deficits	0.365	0.069	5.278	< 0.001***

Response variable: met and unmet needs based on CANE. Explanatory variables: pathologies and sociodemographic variables.

In this case, the following variables are considered as significant predictors of the total number of met or unmet needs:

• Depressive symptoms and cognitive impairment/dementia, as is the case of the model for unmet needs.
• Respiratory pathologies and urological and sensory problems increase the number of met and unmet needs.

### 3.5 Main results

The main results are as follows:

1. People with cognitive impairment/dementia have a greater number of needs (total and unmet) compared to the healthy subsample population.
2. The severity of cognitive impairment/dementia is a significant predictor on both dependency scales (Barthel Index and Lawton and Brody Scale), which are good indicators of the level of dependency in older persons. The more severe the cognitive impairment, the higher the level of dependency. It has been shown that this severity is a predictor of the number of needs, both unmet and total.
3. In the case of total needs there is a relationship with home care, nutrition, personal care, activities of daily living, memory, mobility, incontinence, distress (anxiety) and financial management. In the case of unmet needs, home care, memory, physical health and financial management are related.
4. Living with depression, cognitive impairment/dementia and urological pathology are predictors of having a greater number of unmet needs. Living with depression, cognitive impairment/dementia, a respiratory pathology, a urological pathology and sensory deficits are predictors of having a higher number of total needs.

## 4 Discussion

The aim of the present study was to show the importance of studying needs as part of the comprehensive psychogeriatric assessment of persons with cognitive impairment/dementia and how increased needs, in particular unmet needs, are related to the level of cognitive impairment and dependency.

The most frequent needs found in the study sample are related to physical health (85.5%), visual and auditory deficits (40.9%), distress and anxiety (37.1%) and the state of the home (36.5%), relating in general to the domains of self-care and the physical and psychological sphere. Variable results have been found in other studies, although most are related to the physical, psychological and environmental spheres (social variables). This is the case of the study conducted by Tiativiriyakul et al. (28), that of Hoogendijk E et al. (29) and that of Magalhaes Sousa R et al. (30).

With respect to unmet needs in the study conducted, the most frequent were those pertaining to the psychological and social sphere, with particular emphasis on needs related to companionship, financial management, mobility, distress/anxiety and activities of daily living. The same result was also found in other studies (Titiviriyakul P et al. (28) Passos et al. (31).

Regarding the most frequent unmet needs in people with cognitive impairment, the study found them to be memory, home care, financial management and physical health. In their research based on the Actifcare Cohort Study, Gonçalves-Pereira M et al (32) reported that unmet needs were mostly related to companionship, stress/anxiety and activities of daily living. These results were also found in the study by Mazurek et al (33), as well as in the study by Tobis S et al (34), Kerpershoek K et al (35) and Miranda-Castillo C et al. (11, 36).

The most frequent total needs (met and unmet) in our study of people with cognitive impairment were home care, personal care, financial management, nutrition, mobility, memory, anxiety/stress, activities of daily living, and continence. In the study conducted by Bohlken J et al (37), psychological disorders (related to mood, anxiety/stress), limitation in activities of daily living and memory disorders were highlighted. In their study, Tapia-Muñoz T et al. (38) described home care, nutrition and self-care as the most frequent needs.

In the study carried out by Van der Ploeg ES et al (39), comparing the needs of people over the age of 65 with and without dementia living in a nursing home, it was found that the most frequent needs were those related to housing, financial management, continence, medication, memory, risk of (accidental) self-injury, companionship and activities of daily living (as in our study). This result was the same as that reported by Worden A et al (40) or the study by Hancock G et al (41).

In the present study, one of the main results is the relationship between the diagnosis of cognitive impairment/dementia and needs, finding that they are related to an increase in the number of needs (both met and unmet) and an increase in dependency. Furthermore, the severity of the cognitive impairment has been shown to increase needs (both met and unmet).

The relationship between having cognitive impairment/dementia and having a higher number of needs has been found in most of the literature reviewed (34, 39, 42, 43). However, no such relationship was reported in the study by Ballard C et al. or that of Ashaye OA et al. (44, 45).

The severity of cognitive impairment/dementia is related to an increase in the number of needs (both unmet and total) (31, 43)

There are three characteristics that should be highlighted within the study of the needs of people with a dementia diagnosis, which were also found in our study (although they have not been contrasted in this paper, since only the perspective of the professional has been used within this reanalysis of data):

- People with cognitive impairment report fewer needs as the disease worsens and deterioration progresses (46).
- The perspective of the professional, the caregiver and the person gradually differs as the disease progresses, as a consequence of the point described above (43–48). By contrast, in the study conducted by Orrell M et al. (49), this difference was not statistically significant.
- The caregiver’s age, high caregiver burden and high levels of anxiety are also factors related to the increased needs of people with dementia (35, 47, 48).

The main conclusion of our study is that the use of an instrument that allows the analysis of a large number of biopsychosocial needs, such as CANE, provides essential information for a comprehensive psychogeriatric assessment of the person with dementia and will facilitate the implementation of personalized care, as recommended in virtually all good clinical practice guidelines for psychogeriatric and, more specifically, for dementia care (2, 3, 7–10).

### 4.1 Strengths and limitations

In concluding this discussion, it is essential to consider both the strengths and limitations of the present study, as these factors provide a balanced perspective on the findings and their applicability.

A potential weakness is the limited geographical diversity of the sample. The study was conducted in a single region (Santiago de Compostela, in the Autonomous Region of Galicia, Spain), which might limit the generalizability of the findings to other populations with different demographic and cultural characteristics. Certainly, the conclusions of any epidemiological survey are limited to the social and cultural context in which the data were generated and, therefore, this study should be repeated in other parts of Spain, other European countries and in other contexts that are geographically and culturally more distant. On the other hand, we believe that the study has coherence enough to encourage other research groups to participate in such a collaborative project.

That said, we feel it is important to emphasize that although the territorial location is relatively limited, this is a field study on a representative sample of the general population. Numerous studies of this nature have been conducted and continue to be conducted with data from populations receiving health and/or social care, often in specific care facilities (memory units, day centers, nursing homes, etc.). Our study analyzes a representative sample of the “Santiago de Compostela-Barbanza Health Area”, which is an administrative division of a population that all receives health and social care from the same health and social services. The merit of this Health Area is that it brings together all the socio-economic and cultural nuances of the Autonomous Region of Galicia. In short, we consider that the fact that this is a field study and the socio-cultural richness of the sample are part of the study’s strengths.

Another potential weakness of the study is reliance on outdated data. The study is based on the reanalysis of data from an epidemiological study conducted two decades ago, which might affect the relevance of the findings in the current context. Without a doubt, the needs of the population change over time, as a consequence of advances in knowledge and in social protection systems (although they may also regress), and of other social and political changes, for example. But our study is not a descriptive study of the prevalence of the current needs of older persons in Galicia (although it was at the time). The aim of this study is to analyze the structure of the concept of needs, using data to demonstrate something that is conceptually reasonable to expect: that the study of needs is not opposed to or an alternative to clinical diagnoses or functional assessment, but rather it adds another layer of knowledge about the health status of the older population and helps professionals to make better clinical decisions and social interventions. Due to the fact that the study analyzes the relationship between different types of variables: diagnoses of mental and somatic disorders, functionality, and formally assessed needs, we consider that the age of the sample does not constitute an impediment. This could have been the case if this analysis had been performed on a very specific sample of patients (for example, health care services that no longer exist or whose functioning have been greatly modified over two decades). But we consider that this analysis is valid due to the fact that it is a community sample that is representative of the general population of Galicia. While many things have changed in the past two decades in this territory, we believe that the sociological and healthcare structure and social resources have not experienced radical changes.

Another limitation could be the cross-sectional design of the study. While longitudinal studies on how needs change over time will undoubtedly provide a greater wealth of knowledge, we consider that this kind of analysis deserves to be known and disseminated, among other reasons, to encourage other groups to conduct such longitudinal (preferably international-collaborative) studies.

One of the strengths of the CANE, the instrument chosen to measure needs, is that it includes (in three separate columns) the perspective of the user and the caregiver, as well as the perspective of the professional, making it favorable over other instruments that measure needs. In other words, the caregivers of all the dependent older persons in our study provided insight into the needs of these individuals. Likewise, users always provided their own perspective of their needs, except in cases of dementia so advanced that it prevented dialogue with the patient (which marks the natural limitation of collecting the user’s perspective of their needs). In all cases, with the information provided by the subject and the caregiver (where applicable), the research team drew up the professional opinion on the needs of that older person (i.e., the epidemiological study has mimicked the use of CANE in routine clinical practice).

It seems appropriate to point out that the field study that generated these data was one of the first in the history of CANE, if not the first, to be used in an epidemiological survey (14). As indicated in the brief description of the methods used to prepare the study sample, it includes not only subjects with all levels of severity of cognitive impairment and physical dependence, but also “healthy” subjects (regarding cognitive impairment, depression, Basic Activities of Daily Living and Instrumental Activities of Daily Living). This enables a holistic and detailed analysis of the relationship between needs and the successive stages of cognitive impairment.

## Data Availability

The original contributions presented in the study are included in the article/supplementary material. Further inquiries can be directed to the corresponding author.
